# Oral dysbiosis: methodological evolution, the mobile resistome and the future of machine learning in dentistry

**DOI:** 10.1080/20002297.2026.2717773

**Published:** 2026-08-24

**Authors:** Jack Lynch, David Bradshaw, Ricarda Hawkins, Rob Howlin, Sue Pavitt, Thuy Do

**Affiliations:** a Division of Oral Biology, School of Dentistry, University of Leeds, Leeds, United Kingdom; b Haleon plc, Weybridge, United Kingdom

**Keywords:** Microbiota, drug resistance, microbial, resistome, gene transfer, horizontal, biofilms, whole genome sequencing

## Abstract

The oral microbiome acts as a significant, yet often overlooked, reservoir for antimicrobial resistance genes (ARGs). This review examines the role of the human resistome in oral health, evaluating current knowledge on the composition, function and dissemination of ARGs within the oral cavity. By comparing conventional culture-based methodologies against recent whole-genome sequencing (WGS) studies, we assess the critical role of the oral‒gut axis in resistance dissemination. The current literature indicates that horizontal gene transfer (HGT) facilitates the spread of mobile genetic elements within oral biofilms. Crucially, oral commensals, such as *Fusobacterium spp.*, demonstrate the capacity to disseminate systemically, potentially transferring ARGs to pathogenic species. Recent WGS data further indicates a higher prevalence of ARGs in healthy individuals compared to those with dental caries, alongside an increased potential for ARG mobilisation with age. Ultimately, the dynamic oral resistome plays a vital role in both local and systemic health. Advanced methodologies, including WGS and machine learning, are essential for accurate resistome profiling and predicting antimicrobial susceptibility, which will guide prudent antibiotic prescribing in dentistry and mitigate the spread of antimicrobial resistance.

## Key messages

- The human oral cavity acts as a significant reservoir for antimicrobial resistance genes (ARGs), with oral biofilms providing an ideal environment for horizontal gene transfer.
- Dysbiosis of the oral microbiome not only drives local pathologies like caries and periodontitis but also facilitates the systemic dissemination of resistant pathogens, emphasising the need for dental antibiotic stewardship within a One Health framework.
- Advancing oral microbiome research requires transitioning from descriptive studies to predictive models by integrating conventional methodologies with machine learning and computational biology.

## Introduction

The human body is often described as a complex holobiont, an ecosystem comprising host and microbial communities, including bacteria, fungi, viruses, archaea, and protists [1,2]. These microbial communities aid in host functions such as nutrient metabolism, immune homeostasis and defence against pathogens, playing a crucial role in supporting the physiology and health of the host [3]. Within the human microbiome, individual niches offer unique environments for specific populations of microbes [4].

One of the most important of these niches is the human oral microbiome (HOM). It is recognised as a dynamic and complex environment, second only to the gut in terms of both microbial diversity and density [5]. The oral cavity comprises multiple niches, each with distinct characteristics that support different microbial communities. A balanced oral microbiome contributes to pathogenic colonisation resistance, preventing dysbiotic shifts associated with disease [6]. The oral microbiome's complex interactions and metabolic activities suggest that it functions as an integrated system vital to host health [6]. Key sites within the mouth, such as the teeth, tongue, palate, and cheeks, offer differing factors, including salivary flow, dietary pH changes, and environmental exposure [7]. These factors support the rich microbial community in the oral cavity. For instance, just 1 mL of saliva contains approximately 1 × 10^8^ colony forming units (CFUs), with over 800 different taxa identified in the Human Oral Microbiome Database (HOMD), more than 500 of which primarily colonise the oral cavity [8,9]. While viruses are the most numerous entities in the oral cavity, bacteria dominate the HOM in terms of biomass, particularly phyla such as *Firmicutes, Bacteroidetes, Proteobacteria, Spirochaetes,* and *Fusobacteria*, which together account for about 96% of the observed taxa [10]. However, their composition changes through health and disease, with some more associated with one than the other. Although bacteria make up the majority of the biomass, other organisms, including fungi, archaea, and viruses, are also essential for maintaining balance in the microbial environment. Fungi, primarily *Candida* species, constitute a smaller fraction but exist commensally in health [11,12]. Archaea, like methanogens such as *Methanobrevibacter oralis*, are present in lower numbers and are generally considered non-pathogenic in healthy individuals, although their role in disease is under investigation [13,14]. Finally, the oral virome contains eukaryotic viruses (e.g. Herpesviridae) and bacteriophages, which can shape bacterial communities with infection and lysis [15].

Microbial communities in the mouth are often found within biofilms, commonly known as dental plaque, with the formation of oral biofilms following a well-documented process [16] (Table 1). Early coloniser species, such as *Streptococcus* species, are the first to adhere to surfaces covered by a salivary pellicle, a layer formed by the aggregation of salivary proteins [17]. These early colonisers are vital, as they initiate the production of extracellular polymeric substance (EPS) [18]. Subsequently, secondary colonisers attach via coaggregation, a specific cell-to-cell recognition process that often involves lectin–carbohydrate adhesion. *Fusobacterium nucleatum* acts as a key bridging organism, facilitating the binding of late colonisers, often Gram-negative anaerobes such as *Porphyromonas gingivalis* [16,19]. Throughout this process, EPS surrounds microbial cells, enhancing the structure of the biofilm and promoting cell-to-cell adhesion. The growth of biofilms offers significant advantages over planktonic cells, as they protect microbes from host immune responses, antimicrobial agents, and mechanical forces such as tooth brushing [20].

**Table 1.** Oral species and their key characteristics by abundance.

Phylum	Key genera	Gram stain (typical)	Oxygen requirement (typical)	General role in health	References
Firmicutes	*Streptococcus, Veillonella, Lactobacillus*	Positive (*Streptococcus, Lactobacillus*), Negative (*Veillonella*)	Facultative/Anaerobic	Primary colonisers, acid production/utilisation, pH balance (some *Strep,*), biofilm structure, competitive exclusion	[21,22]
Proteobacteria	*Neisseria, Haemophilus, Aggregatibacter*	Negative	Facultative/Aerobic	Early colonisers, nitrate reduction (some *Neisseria*) and immune modulation	[23–25]
Bacteroidetes	*Prevotella, Porphyromonas, Tannerella*	Negative	Anaerobic	Low abundance in health, can be pathobionts, involved in complex metabolic networks within biofilms	[26–28]
Fusobacteria	*Fusobacterium*	Negative	Anaerobic	Bridging organism in biofilm coaggregation, structural role, can be a pathobiont	[29–31]
Actinobacteria	*Actinomyces, Corynebacterium, Rothia*	Positive	Facultative/Anaerobic	Primary/secondary colonisers, biofilm formation, metabolic interactions, some contribute to pH homeostasis	[32,33]
Spirochaetes	*Treponema*	Negative (Gram-indeterminate normally)	Anaerobic	Low abundance in health, associated with periodontal pockets, motile	[34,35]

Furthermore, these intrinsic characteristics are essential in the development of dysbiosis [36]. The robust physical barrier of the EPS and metabolic adaptability of the biofilm allow these microbes to endure environmental shifts [37]. The EPS matrix acts as a diffusion-limiting barrier that facilitates acid capturing, trapping organic acids produced during microbial metabolism, lowering the local pH and creating selective ecological pressure that favour the proliferation of acid-tolerant microbes [38,39]. However, this resilience can facilitate the enrichment of acidogenic or inflammophilic species when the host is exposed to risk factors such as frequent dietary sugars or poor hygiene. This resistance to chemical and mechanical disruption helps drive the shift towards dysbiosis [37,40]. Specifically, the structural nature of the biofilm allows microbial communities to be protected from salivary clearance and mechanical forces such as mastication and antimicrobials, allowing the dysbiotic state to mature and persist [40]. In the oral microbiome, dysbiosis is linked to various oral diseases, including dental caries and periodontitis, and is also linked to systemic diseases such as rheumatoid arthritis and cancer [41]. External pressures and changes result in fluctuations in species abundance and diversity in HOM, which may trigger dysbiosis. Factors influencing these shifts include host genetics, diet, oral hygiene practices, antibiotic use, and preexisting systemic diseases [42].

Despite this dynamic nature, the HOM in health is usually resilient, being able to withstand environmental changes and prevent the transition to dysbiosis and maintain a state of microbial balance, known as eubiosis [43]. Several factors from the host are vital for this stability. Saliva, for example, plays multiple roles, including buffering changes in pH, supplying essential nutrients to commensal microbes, and containing antimicrobial proteins and peptides that selectively target pathogens [44]. Additionally, the immune system in the oral cavity is adapted to its environment, employing immunological strategies to manage continuous exposure to various microbes. Central to this system is its ability to differentiate between commensal microorganisms and pathogenic ones, through the use of Pattern Recognition Receptors (PRR) [45]. Though PRRs are not unique to the oral cavity, their ability for distinction is essential for maintaining the health and balance of HOM.

Moreover, complex interactions among microbes, such as synergism, commensalism, and antagonism, are essential for the HOM’s stability and the ability to prevent pathogens' colonisation, through competition for nutrients and the production of antimicrobials [46]. Interestingly, some studies indicate that certain oral bacteria influence the host's taste perception and dietary choices, potentially creating a feedback loop that helps maintain their presence in the mouth [47]. This illustrates a closely linked, coevolved relationship in which the host provides a habitat and nutrients, while the microbial community offers beneficial services. Recognising the various factors from both the host and microbes contributing to this resilience is crucial for developing methods to prevent dysbiosis or restore a healthy microbial balance when disrupted.

## Major oral diseases associated with dysbiosis: dental caries and periodontal disease

Dental caries and periodontal diseases represent two of the most prevalent non-communicable diseases affecting the human population and are closely tied to dysbiosis of the oral microbiome [48]. While both conditions arise from overall dysbiotic oral biofilm formation, they are characterised by distinct disease-associated species, mechanisms, and clinical outcomes (Table 2).

**Table 2.** Caries and periodontitis and their key characteristic.

Feature	Dental caries	Periodontitis	References
Primary etiological driver	Frequent dietary sugar intake leading to prolonged low pH	Host inflammatory/immune response to dysbiotic subgingival biofilm	[49,50]
Key microbial signatures/pathogens	Acidogenic/aciduric: *Streptococcus mutans, Lactobacillus spp., Actinomyces spp*.	Proteolytic/anaerobic: ‘Red complex’ *(P. gingivalis, T. forsythia, T. denticola*), *F. nucleatum, Prevotella spp., F. alocis*	[29,51,52]
Nature of dysbiosis	Shift towards an acid-producing and acid-tolerant microbial community	Shift towards a community thriving in an inflammatory, anaerobic, proteolytic environment	[49,50]
Primary site of biofilm	Supragingival tooth surfaces (enamel, dentine)	Subgingival sites, within the periodontal pocket	[29,53–55]
Key pathogenic mechanisms	Demineralisation of tooth hard tissues by bacterial metabolic acids	Host-mediated inflammatory destruction of periodontal ligament and alveolar bone	[56,57]
Reversibility	Initial lesions (white spots) are reversible, advanced lesions require restoration	Gingivitis is reversible, periodontitis involves irreversible loss of attachment and bone	[57,58]
Major clinical outcomes	Cavities, pain, pulpal infection, tooth loss	Periodontal pocket, bone loss, gingival recession, tooth mobility, tooth loss	[53,57,58]

### Dental caries

As aforementioned, HOM dysbiosis can lead to disease within the oral cavity and systemically. Dental caries involves the demineralisation of tooth enamel caused by a dysbiotic shift toward acidogenic and aciduric bacteria, primarily due to frequent sugar consumption [10,59]. The metabolism of sugars by bacteria produces organic acids, such as lactic acid, which lowers the plaque pH. When the pH drops below 5.5, demineralisation of the enamel begins. Cavities develop if this process persists and outpaces the natural restoration of minerals within enamel, called remineralisation [60].

Key pathogenic bacteria in caries include *Streptococcus mutans*, which is thought to be the primary pathogen due to its ability for sugar metabolism, acid production (acidogenicity), and acid tolerance (aciduricity), and its frequent presence in those with caries [61,62]. *S. mutans* adheres to tooth surfaces through specific adhesins, creating EPS from sucrose [63,64]. This EPS matrix captures acids, resulting in a localised decrease in pH [65]. Mechanisms that confer acid tolerance include proton pumps (F₁F₀-ATPase) and DNA repair systems [66,67]. Other significant species include *Lactobacillus spp.* (linked to lesion progression owing to their high acidogenicity/aciduricity) and *Actinomyces spp.* (particularly relevant in root caries) [68,69]. Moreover, *Scardovia wiggsiae* is often associated with severe early childhood caries, even found frequently in those with caries in the absence of established caries pathogens such as *S. mutans* [70].

The HOM in caries exhibits a transition from health-associated non-mutans streptococci and *Actinomyces* to a predominance of aciduric species, such as *S. mutans*, particularly in initial lesions, with a reduction in overall species diversity [71]. In contrast, dentin caries may exhibit increased diversity compared to enamel caries [71]. Clinically, caries initiates as a reversible white spot lesion. If left untreated, it can progress past the enamel into the dentin, potentially reaching the pulp and causing pulpitis, pain and eventual tooth loss [72].

### Periodontal diseases

Periodontal diseases encompass inflammatory conditions that affect the structures supporting teeth caused by dysbiotic biofilms and host immune responses. Gingivitis, characterised by reversible inflammation of gums with no attachment loss, is driven primarily by non-specific plaque accumulation [73]. In this condition, the microbial community is marked by an increased abundance of Gram-negative anaerobic and facultative species, such as *Prevotella* and *Fusobacterium*, respectively [74]. If gingivitis remains untreated, it can progress to periodontitis.

Periodontitis is a chronic, irreversible inflammatory disease that leads to destruction of the periodontal ligament and alveolar bone, resulting in the formation of periodontal pockets, gum recession, increased tooth mobility, and eventual tooth loss [75]. This arises from the interplay between the dysbiotic subgingival microbiome and a dysregulated immune response in a susceptible host [76]. The microbial profile of periodontitis is characterised by an enrichment of predominantly Gram-negative, anaerobic, and proteolytic bacteria [77]. The ‘red complex’ bacteria *P. gingivalis, Tannerella forsythia,* and *Treponema denticola*, are strongly implicated in its pathogenesis [78]. These are labelled ‘red complex’ pathogens, as they represent the climax community of periodontal infection, often resulting in the highest risk for severe tissue destruction and tooth loss when highly enriched. Additional key species include *F. nucleatum, Prevotella intermedia, Aggregatibacter actinomycetemcomitans,* and the emerging periodontal pathogen *Filifactor alocis* [79,80].

The pathogenesis of periodontitis involves various virulence factors from these microbes. *P. gingivalis*, identified as a keystone pathogen, a pathogen in low abundance that can initiate pro-inflammatory responses, drives dysbiosis and proliferates in pro-inflammatory environments [81,82]. Crucial to its pathogenesis are *P. gingivalis's* gingipains, which degrade host proteins, impair neutrophil function, and disrupt epithelial barriers, while *P. gingivalis* LPS promotes chronic inflammation by activating host immune cells [83,84]. Additionally, *T. denticola* utilises motility for tissue penetration, major outer sheath protein (Msp) for adhesion and pore formation and dentilisin (CTLP protease) for tissue degradation [85,86]. *F. nucleatum* functions as a coaggregation bridge, with the FadA adhesin facilitating host cell binding and invasion and enhancing endothelial permeability by disrupting cell‒cell junctions [87]. Lastly, *F. alocis* demonstrates adhesion and invasion capabilities, resists oxidative stress, and produces proteases that may contribute to the overall pathology of periodontal diseases [79].

## Discussion

### The oral resistome and mobilome: emerging interest

In addition to its taxonomic interest, HOM has highlighted the systemic and environmental impacts of oral microbes, including the dissemination of antimicrobial resistance (AMR). The problem of AMR is becoming an ever-growing threat to public health, with an estimated 10 million deaths per annum associated with AMR by 2050 [88]. Additionally, it causes increased healthcare costs, demands, and lost work hours due to complications in treating previously treatable infections. The primary driver of this crisis is the misuse of antimicrobials, not just in human healthcare but also in veterinary medicine and agriculture [89].

The term resistome includes all antimicrobial resistance genes (ARGs), as well as their precursors, that are found within a given microbiome [90]. ARGs' primary function allows microbes to survive antibiotics in the environment through numerous mechanisms, such as target alteration or antibiotic metabolism, against environmental antibiotics [91]. Although typically referred to in the context of healthcare, these mechanisms are often a result of the inherent defence of microbes against interspecies warfare with other microbes [92]. The evolution of the resistome is significantly influenced by HGT, the spread of mobile genetic elements (MGEs), both intra- and inter-species. The rate of HGT is further enhanced by antibiotics and environmental pressures [93]. Therefore, it is essential to monitor this development, to understand how ARGs transfer between different ecological niches [94].

Resistomes are ubiquitous across diverse environments, including soil, water systems, agriculture, and the complex microbiomes of animals and humans, such as the gut and oral cavity [95,96] Environmental microbiomes are considered reservoirs where ARGs can transfer to human pathogenic bacteria [97,98]. Local ecological conditions and selective pressures influence the prevalence and variety of ARGs. These local resistomes are linked through microbial dissemination and subsequent HGT, supporting the idea of a ‘meta-resistome’ [99]. Additionally, environmental pollution, including antibiotic residues and heavy metals, can directly influence the evolution and spread of resistance by increasing HGT rates [100].

Acquired resistance occurs via several mechanisms, including limited drug uptake, reduced porin channels or biofilm formation, modification of drug targets, such as changes in antibiotic-binding proteins or ribosomal components, drug inactivation, for instance, via β-lactamase enzymes that break down penicillin, and active efflux of drugs through membrane pumps [101]. The primary mechanism for the spread of acquired ARGs is HGT, which includes transformation (uptake of free DNA), transduction (DNA transfer via bacteriophages), and conjugation (direct DNA transfer between cells) [102,103]. MGEs, such as plasmids, transposons, integrons, and bacteriophages, enable HGT [104]. MGEs can also carry other adaptive genes, such as those associated with virulence. However, carrying these elements often incurs a biological cost, resulting in reduced fitness in the absence of selective pressures [105,106]. Despite this cost, selection for antibiotic resistance might additionally boost pathogenicity, as demonstrated in oral streptococci. [107].

The oral cavity serves as a significant reservoir for ARGs, constituting the oral resistome [108]. The oral mobilome comprises MGEs, including plasmids, transposons (e.g. Tn*916*), integrons, and bacteriophages. [109]. Oral biofilms are vital for maintaining and spreading the oral resistome. The high microbial density and closeness within biofilms create an optimal microenvironment for HGT [110,111]. Crucially, oral bacteria and their ARGs are not limited to the mouth and can spread systemically, contributing to infections elsewhere and the wider AMR challenge [112].

For example, a systematic review identified 159 distinct ARGs conferring resistance to 22 different antibiotic classes across various oral sites, with saliva and supragingival biofilms showing the greatest ARG diversity. Common oral resistance genes include those against macrolides, for example, *ermB*, *msr(D),* and *mef(A)*, tetracyclines, such as *tet(M*) and *tet(O),* and beta-lactams, such as *blaTEM* [108]. Adapted from this review, Table 3 gives a detailed summary of these major ARG classes commonly found in the oral cavity. Further investigation has identified a core oral resistome across different oral sites, with three ARGs, *tet(M), tet(O),* and *ermB*, found consistently across all the sampled oral sites, indicating that they form a fundamental component of the oral resistome [108]. Interestingly, previous findings suggest that a healthy oral microbiome might paradoxically contain a richer resistome at the antibiotic class level compared to caries-affected children [113]. This richness likely reflects a highly diverse, commensal microbial community where ARGs naturally occur as mechanisms of microbial competition rather than as a direct threat to treatment. However, when dysbiosis occurs and specific pathogens dominate, the accumulation and mobilisation of these ARGs within a disease-associated community can significantly reduce the effectiveness of dental treatments for conditions such as periodontitis and dental abscesses [114,115].

**Table 3.** Antibiotic classes and their corresponding genes and implications.

Antibiotic class	Common ARG examples	Associated MGE types	Implicated bacterial genera (examples)	Potential clinical significance	References
Tetracyclines	*tet(M), tet(O), tet(Q), tet(W)*	Transposons (e.g. Tn*916*-family ICEs), Plasmids, IMEs	*Streptococcus, Actinomyces, Veillonella, Fusobacterium*	Resistance to commonly used broad-spectrum antibiotics in medicine and dentistry	[108,116]
Macrolides, lincosamides, streptogramins (MLS)	*erm(B), erm(F), mef(A), msr(D)*	Plasmids, Transposons, ICEs, IMEs	*Streptococcus, Actinomyces, Staphylococcus*	Resistance to necessary antibiotics for respiratory, skin and oral infections	[108,117]
Beta-lactams	*blaTEM, blaCTX-M, mecA*	Plasmids, Transposons	*Streptococcus, Neisseria, Haemophilus*, ESKAPE pathogens	Resistance to penicillins, cephalosporins, and other beta-lactam antibiotics, critical AMR concern	[108,118]
Aminoglycosides	*aac(6′)-aph(2″), aph(3′)-IIIa*	Plasmids, Integrons	*Enterococcus*, Gram-negative bacteria e.g. ESKAPE pathogens	Resistance to antibiotics used for serious Gram-negative and some Gram-positive infections	[108,119]
Fluoroquinolones	*qnr genes, mutations in gyrA/parC*	Plasmid-mediated (qnr), Chromosomal mutations	Various Gram-negative and Gram-positive bacteria	Resistance to broad-spectrum synthetic antibiotics used for a variety of infections	[108,120,121]

Within the oral mobilome, conjugative and chromosomally integrated MGEs (cciMGEs), including integrative and conjugative elements (ICEs) and integrative mobilisable elements (IMEs), self-transferring and MGEs that require mobilisation, respectively, are common [105,122–124]. These are particularly ubiquitous in oral streptococci and often co-carry ARGs such as *tet(M)* and *ermB* alongside virulence factor genes [107]. The Tn*916* transposase, an ICE, is correlated with ARGs in the developing paediatric oral resistome, and this potential for mobilisation seems to increase with age [113]. These carry a diverse cargo of accessory genes that provide broader selective advantages to the host, including ARGs and fitness-improving genes, such as tolerance to heavy metals and production of bacteriocins, building host resistance and enhanced survival [125–127]. Tn*916*-family ICEs are particularly significant because they possess a broad host range, allowing them to transfer resistance determinants across different bacterial genera, thereby acting as a major mechanism for ARG dissemination in the oral cavity [107,109,113,126]. As they physically link multiple traits on a single MGE, the HGT of Tn*916*-like ICEs can transform a susceptible commensal bacterium into a resistant strain. While the acquisition of these elements can impose a significant fitness cost on the host, such as reduced growth and metabolic function, recent research has demonstrated that oral species such as *Streptococcus oralis* can undergo rapid evolutionary adaptation to mitigate these costs [109]. Within 500 generations, the host restores its growth rate and reallocates resources to maintain the ICE for long-term competitive advantage. This persistent metabolic shift highlights how these elements drive the evolution and transferability of the oral microbiome, ensuring that once acquired, these resistance traits become stably integrated into the oral microbial community [109].

The growing resistance to common dental antibiotics is a significant concern. This issue is compounded by mechanisms such as exposure to subinhibitory antibiotic doses. Rather than eliminating bacteria, these low concentrations promote a selective pressure that can enrich for resistant strains and remove competition from susceptible microbes, allowing them to establish within the niche [128,129]. Additionally, they can promote ARG formation by inducing error-prone DNA polymerases [130]. It is important to note that resistance is also acquired through chromosomal mutations and vertical gene transfer. Induced error-prone polymerases can cause mutations that alter existing DNA, such as modifying the target site of an antibiotic, leading to reduced drug binding and increased resistance [131,132]. Low-level antibiotic exposure is a particular problem in healthcare settings, including dentistry, where antibiotics may be used prophylactically at low doses [133]. Consequently, prescribing antibiotics in dentistry without prior susceptibility testing can result in ineffective treatment and the progression of dental infections [115].

The inherent tolerance of microbial biofilms to antimicrobials further exacerbates this, as AMR organisms increase resistance and often require more invasive treatments, such as mechanical debridement [133]. Despite this, dental practitioners in the UK account for approximately 10% of all antibiotic prescriptions [134]. These practices, particularly the overuse of broad-spectrum antibiotics such as amoxicillin, increase antibiotic exposure in the oral cavity, exerting significant selective pressure and promoting the survival and proliferation of resistant microbial strains [135]. Interestingly, while amoxicillin is the most frequently prescribed antibiotic in dentistry, the core oral resistome is dominated by genes conferring resistance to tetracyclines and macrolides [113]. This discrepancy may arise because tetracyclines and macrolide resistance genes are often co-located on MGEs such as Tn*916*, allowing them to persist and spread effectively, even in the absence of direct selective pressures, or as a result of cross-selection from broad-spectrum antibiotic usage. For these reasons, dental antibiotic stewardship programs are essential [136].

Furthermore, the oral cavity is not an isolated system. Oral microbes can disseminate to other parts of the body and the wider environment. Oral microorganisms can enter the bloodstream through breaks in the oral mucosal barrier, which can occur with conditions such as periodontitis, during invasive dental procedures such as extractions or scaling, or even through daily activities such as tooth brushing, particularly when gingival inflammation is present [137,138]. Additionally, continuous saliva swallowing provides a direct route for these microbes to reach the gastrointestinal tract. While the acidic environment of the stomach serves as a formidable barrier, oral bacteria, particularly those encased in protective biofilms or food matrices allowing them to survive gastric transit [139]. Consequently, around 35% of classifiable oral microbes are able to colonise the gut, contributing to at least 2% of the identifiable microbes in faeces [140].

The spread of oral bacteria to other body sites has been implicated in various systemic diseases, including cardiovascular issues such as infective endocarditis, inflammatory bowel disease (IBD), rheumatoid arthritis and certain cancers [112]. ARG-carrying oral bacteria can make systemic infections more challenging to treat, with documented cases, such as oral streptococci linked to infective endocarditis [141]. MGEs worsen this risk by enabling the spread of ARGs between microbial communities and environmental microbes [107–109]. This potential for oral bacteria, ARGs and MGEs to disseminate systemically, coupled with the continuous exchange of microbes between humans, animals and the environment, firmly places the oral resistome within the One Health framework. Examining all resistomes across these interconnected areas is vital for understanding AMR transmission, creating effective surveillance systems and guiding antibiotic stewardship policies [113]. While the One Health framework provides a conceptual structure for understanding resistome dissemination, direct evidence linking specific oral-derived ARGs to environmental antibiotic selection for human clinical outcomes remains limited and warrants further longitudinal investigation.

## Studying oral dysbiosis: methodological approaches and their limitations

To investigate the influence of HOM and its connection to disease, there has been a need for new methodologies, and it has benefited significantly from the ongoing development from conventional culture-based methods to more complex *in silico* analyses [142]. However, a common theme is their inherent limitations, meaning that no single approach is perfect and must be carefully considered when designing study protocols, underlining the need for multifaceted approaches.

### Conventional culture-based approaches

Conventional microbiology relied on culture-based methods to understand microbes within the human body, including HOM. Culture-based methods encompass a wide range of protocols, from using non-selective and selective media for microbial growth to developing anaerobic techniques for isolating anaerobic microbes from the oral cavity [143,144]. These allowed for early studies to develop an understanding of oral bacteria's morphological, physiological and biochemical characteristics, as well as the susceptibility of isolates to antimicrobials [145]. These techniques were crucial for identifying many of the microbes we now know as oral commensals and pathogens and provide the basis for oral microbiome research [146,147].

Despite the rise of sequencing, culture-based approaches remain indispensable. They are essential for isolating viable strains to conduct downstream phenotypic validation, formulate probiotics and test targeted antimicrobial therapies. Culturing allows direct antimicrobial susceptibility testing (AST), which is critical for understanding the functional capacity of microbiomes. Furthermore, only cultured isolates can be used in advanced *in vitro* and *in vivo* infection models of oral microbiome dysbiosis.

However, these approaches have many significant limitations. One of the most important limitations is the number of unculturable microbes harboured in the mouth, with estimates that around 34% of oral bacteria cannot be cultured using standard protocols [43,148]. This is primarily attributed to the fastidious nature of these microbes, requiring specialised media and growth conditions that cannot be easily replicated *in vitro* [6]. Consequently, culture-dependent studies of the HOM provided a biased and incomplete representation of microbial diversity and composition.

Importantly, when deciphering the role of specific microbes in health and disease, the phenotypic expression of these organisms is essential, enabling researchers to identify potential virulence factors and assess host risk. However, the isolation and growth of microbes in conventional planktonic monocultures can alter these phenotypes, often leading to the expression of genes different from those found *in vivo*, especially those commonly associated with biofilm formation [149]. This relates to the lack of a community context, which reduces the complexity of microbiomes to a single dimension [150]. This reduction limits the ability to study inter-species interactions, such as synergistic or antagonistic relationships, as well as the structure of biofilms formed, all of which are crucial for understanding the implications of oral biofilms in health and disease [151]. Finally, these conventional methods are often labour-intensive and time-consuming, reducing the scalability for high-throughput analysis [152]. The limitations of culture-based methods, particularly the inability to capture the full extent of microbial diversity, directly motivated the development and widespread adoption of culture-independent molecular techniques [8].

### Molecular and sequencing-based approaches

The acknowledged limitations of conventional culture-based methods necessitated the development and refinement of culture-independent techniques. Molecular approaches, which primarily target microbial nucleic acids (DNA and RNA), have revolutionised the study of the oral microbiome. Key target regions such as the 16S ribosomal RNA (rRNA) gene for bacteria and archaea or internal transcribed spacer region for fungi, allow for sequencing for the assessment of microbial diversity and community composition [153]. Shotgun metagenomics provides a further advancement by sequencing all DNA present in a sample, providing insights not only into taxonomic composition but also into the collective functional potential of the community by identifying genes encoding metabolic pathways [154]. Alongside these DNA-based approaches, metatranscriptomics, which involves sequencing RNA, has allowed for the study of gene expression within the community, indicating that active gene pathways within microbiomes, rather than just insights into potential functions given by DNA sequencing [155].

The increased availability of whole genome sequencing, as well as the establishment of digital databases for curation of these sequencing data, such as the Sequencing Read Archive (SRA), European Nucleotide Archive (ENA) and the HOMD, has led to a significant increase in the number of studies moving away from conventional lab-based methodologies to *in silico* techniques [156,157]. The creation of these repositories has made research easier for researchers. For example, the HOMD, established by Dewhirst et al. (2010), provides a curated, oral-specific database of 836 unique species, 525 of which primarily colonise the oral cavity (as of Q3 2026). It is based on 16S rRNA gene sequences, offering genomic information for each and analytic tools for other researchers to use, as well as a repository for new studies to upload their data. This standardisation of the HOMD has been crucial for comparing key findings across studies, leading to improved hypothesis generation and biomarker identification.

For example, Wang et al. (2025) performed a meta-analysis of 1,255 samples from oral squamous cell carcinoma (OSCC)-related 16S rRNA gene datasets. These were from the SRA and ENA databases and exclusively utilised the Illumina MiSeq sequencing platform. They showed that in biopsy samples, the microbiomes of cancerous tissues and adjacent normal tissues were more similar to one another than those of fibroepithelial polyps. They identified key genera and pathways to develop a diagnostic model for OSCC using random forest algorithms on swab samples, with an AUC of 0.918 within these samples and 0.849 when compared to data gathered from the NextSeq platform [158].

This demonstrates the potential for using publicly available data for large-scale cohort analysis, reducing the effect of localised variables such as diet while providing a new scope for analysis, including correlations that the primary researcher did not previously conduct, such as those related to the resistome and mobilome. However, Wang et al. noted a discrepancy in model performance between sequencing platforms. While the platform itself plays a role, such differences are multifactorial. Inherent variations between independent datasets, including differing patient demographics, DNA extraction techniques, and sampling methodologies, introduce batch effects that can heavily confound machine learning models and cross-study comparisons.

Furthermore, sequencing-based approaches carry their own limitations. Shotgun metagenomics, while comprehensive, struggle with host DNA contamination, which is particularly prevalent in saliva and oral tissue samples, leading to reduced microbial sequencing depth. Additionally, metagenomics can also struggle to distinguish between live and dead microbes without the additional use of viability dyes, potentially skewing the representation of the active microbiome. Crucially, when studying the oral resistome and mobilome using short-read sequencing data, it is difficult to confidently assemble MGEs or link specific ARGs to their host bacterial genome, meaning that it is possible to identify ARGs present but not the carrying microbe.

Despite this, publicly available data from published sources and databases is challenging to access. Many publications demonstrate suboptimal quality and standardisation of associated metadata. Kim et al. (2025) found that within gut microbiome studies, nearly half of the reviewed 3000 studies did not meet the minimum standard for sequence data availability and had poor metadata, lacking key descriptors such as host age and sex for individual samples [159]. This creates a barrier for those wanting to re-analyse data, as key comparisons are unable to be made due to the lack of transparency and repeatability in these studies, which limits confidence in cross-study comparisons [160]. Additionally, as with the work of Wang et al. (2025), comparisons made between different sequencing platforms may not be fully comparable. However, there are still apparent differences, as quantified by the reduction in confidence when deploying the same random forest algorithm. This is controllable by selecting datasets obtained from only the same sequencing platform, but this limits the cohort size. At the same time, other parameters are less easily controlled and selected for. For example, DNA/RNA extractions, while similar in methodology, are heavily dependent on the researcher performing them [161]. Similarly, when collecting patient samples, the resulting quality and composition of the microbiome data are heavily dependent on the participant and the researcher involved [162]. These confounding variables can influence the results and provide a skewed representation of the true HOM.

Furthermore, whilst the availability of this data is of great importance and usefulness to the oral microbiome research community, resources such as high-performance computer nodes greatly limit the plausibility of large-scale data sets [142]. Additionally, bioinformatic pipelines require expertise that is not readily apparent to most, with a multitude of pipelines available and little homogeneity between studies, which hampers the ease of utilising this data and their comparability once analysed [142,163].

### Experimental models

While sequencing-based approaches provide a comprehensive cross-sectional overview of taxonomic and genetic potential in both cross-sectional and longitudinal study designs, experimental models are essential for mechanistic analysis of interactions with the host, contributing to the understanding of dysbiosis development between health and disease.

#### *In vivo* models

*In vivo* studies of the oral microbiome encompass both human and animal studies, allowing insights into real systemic host responses. However, they come with significant scientific and ethical hurdles.

Animal models, including rodents, dogs and primates, have been particularly effective in periodontal and caries research. A key example of this is within caries research. Jiang et al. (2023) were able to utilize rat models to demonstrate the ability of *S. mutans* UA159 to proliferate after inoculation with a three-day high-sucrose diet, resulting in damage to deep dentin layers [164]. Animal models, often constructed by inoculating a single pathogenic organism, have allowed researchers to study specific aspects of oral disease and the effects of individual species on disease progression in live hosts [165]. These models allowed contextualising host immune responses and therefore the efficiency of targeted interventions, which would be impossible to test directly in humans. However, their use is significantly constrained by several factors. For example, the cost of maintaining these models is significant, requiring specialised animal housing facilities, dedicated veterinary care and time spent adhering to stringent ethical review processes. Ethical practices in animal models are becoming increasingly pressing, with initiatives such as the 3Rs (Replacement, Reduction, and Refinement) placing pressure on researchers to move away from animal testing where possible [166]. More importantly, there is a significant difference in the makeup of the oral microbiome across host species, limiting the direct applicability of targeted interventions and findings in these models [167,168]. Additionally, the common practice of using a single defined species to represent polymicrobial diseases reduces their complexity and cannot accurately reflect the intricate nature of human oral diseases [165]. Furthermore, as with any *in vivo* study, there is greater inter-individual variability than *in vitro* studies. This variability not only makes it difficult to fully standardise experiments, but also necessitates sufficiently large sample sizes to achieve the statistical power required to understand the roles of specific microbial or host factors in disease development [169].

In addition to animal *in vivo* studies, human studies have been conducted, and by their nature, they offer direct relevance to human health and disease. Observational studies have played a crucial role in identifying associations between the microbiome and oral and systemic clinical diagnoses. For example, Al-Maweri et al. (2025) conducted a systematic review of real patient samples from the oral microbiome and hypertension and discovered a significant difference in the oral microbiome taxa between those who were hypertensive and normotensive, despite heterogeneity between the studies [170]. Some microbial taxa, such as *Prevotella* and *Veillonella*, were consistently enriched in individuals with hypertension, as observed in studies that controlled for false discovery rates. Similarly, connections have been established between oral cancer, OSCC and colorectal cancer through studies conducted within the human oral cavity, providing substantial evidence for the impact of the oral microbiome on systemic diseases [41]. These have provided essential information for generating hypotheses on the importance of oral health and the effects of poor oral hygiene, both locally and systemically.

However, these studies also face limitations. Similar to animal models, stringent ethical review frameworks can limit the plausibility of these experiments, with ethical conflicts arising from inducing oral dysbiosis in otherwise healthy individuals. Nevertheless, not all dysbiotic biofilms need to be induced. With a large cohort, naturally occurring dysbiosis could potentially be captured by research through analysis of patients with varying levels of disease before subsequent treatment, though this approach carries substantial ethical and practical costs. The result is that many of these studies are cross-sectional, focusing on the effects of disease after onset, and are descriptive or correlational, lacking the ability to distinguish cause from effect for oral dysbiosis and its associated diseases. This means that these studies often cannot definitively establish causality without additional mechanistic investigations in controlled environments, as is possible in longitudinal, induced dysbiosis studies [171,172]. Human studies are further limited by their lack of controlled subjects, with variability present between participants' genetics, diets and other factors [173,174]. Whilst inclusion/exclusion criteria for factors such as smoking status or antibiotic use can help reduce these effects, the reproducibility of these studies remains challenging, with studies often requiring large cohorts to control for these effects and revealing true biological significance, thereby increasing the complexity and overall cost of such studies.

#### *In vitro* models

To mitigate the limitations of *in vivo* studies, particularly regarding variable control and repeatability, numerous studies have attempted to recreate the oral microbiome *in vitro* [175,176]. These studies have been invaluable for discovering the pathogenic mechanisms and virulence factors of disease-associated species, such as ‘red complex’ species (*P. gingivalis, T. forsythia,* and *T. denticola*), in controlled environments, providing early insights into their potential role in periodontitis [177]. Additionally, these *in vitro* models have allowed insights into the formation of dental biofilms. Sánchez et al. (2011) created simplified models of oral biofilms using six key oral taxa (*S. oralis*, *Actinomyces naeslundii*, *Veillonella parvula*, *F. nucleatum*, *P. gingivalis,* and *A. actinomycetemcomitans*) grown on hydroxyapatite discs coated in salivary pellicles [178]. They were able to show a pattern of microbial colonisation and biofilm maturation similar to what is observed in subgingival *in vivo* plaque [105]. Specifically showing that early colonisers adhered within 12 h, followed by *F. nucleatum* within 24 h and late colonisers within 48 h. Furthermore, their results showed biofilms reached a steady state between 72 and 96 h, with bacterial vitality increasing between the surface and centre of the biofilm.

Using confocal laser scanning microscopy, they demonstrated biofilm structures, species distribution within biofilms, and key differences between the upper and lower biofilm, illustrating the sequential colonisation of bacteria as biofilms mature. A key aspect of these models, such as the six-species model, compared to *in vivo* models, is the defined consortium, which shows high reproducibility, allowing other researchers to utilise it in various studies, including studies of the effects of antibiotic treatment. These studies demonstrate the utility of conventional models in addressing specific questions about oral biofilms and provide reproducible platforms for further investigation.

However, a major drawback of these models is their significant oversimplification, which limits their ability to reflect the complex nature of the HOM accurately. A clear example of this is their drastic lack of microbial diversity compared to *in vivo* samples, which contain just six key bacteria in the model by Sánchez et al. (2011). In contrast, over 800 species are observed in the oral cavity [148]. Not incorporating non-bacterial species reduces the likelihood of detecting the effects of low-abundant organisms on disease prognosis. This microbial simplification also reduces the ability to study and decipher inter-species interactions such as metabolic cross-feeding, competition and cell‒cell signalling, which are key in all microbiomes, not just the oral cavity.

However, new developments have incorporated the use of patient-derived inoculum for the production of orally relevant biofilm. Naginyte et al. (2019) developed a model to enrich for periodontal pathogens from samples taken from dentally healthy adults by growing pooled saliva, supragingival plaque, and tongue scrapings as a biofilm under conditions simulating an inflamed periodontal pocket. Metagenomic analysis revealed a significant shift from a health-associated microbial profile to one enriched in taxa implicated in periodontitis, such as *P. gingivalis* and *T. forsythia*, supporting the polymicrobial synergy and dysbiosis hypothesis [176]. This study, however, also showed significant differences between the diversity and species present in the samples between the inoculum and after incubation, though crucially this included a number of fastidious organisms commonly found in the mouth that were undetectable in the initial inoculum. This highlights the importance of co-culture experiments, providing a complex community context with shared metabolites and cell-to-cell signalling, allowing for historically unculturable species to thrive *in vitro* alongside their natural microbial cohabitant, suggesting increased representation of the oral cavity and the ability to culture conventionally unculturable oral microbes.

However, the importance of crosstalk should not be omitted, and the model still lacks incorporation of host factors. Within the oral cavity, fluid dynamics play a crucial role in biofilm formation, as saliva and gingival crevicular fluid generate shear forces, buffer the pH and establish nutrient and oxygen gradients. Most *in vitro* studies use static media and conditions for reproducibility. Additionally, studies lack key host factors, such as the absence of host cells in these models, the exclusion of epithelial or immune cells, and the omission of key salivary constituents secreted by these cells, including antimicrobial proteins, mucins, and mechanisms to instigate a host response [179,180]. These host factors are crucial to the formation of dental biofilms *in vivo*, modulating microbial adhesion and pathogenesis, and therefore significantly affect disease outcomes. Nevertheless, the inclusion of these cells also has significant drawbacks for the plausibility of *in vitro* models, as the rapid growth of oral microbes co-cultured with mammalian cells can lead to the accumulation of endotoxins due to prolonged exposure to static conditions, resulting in unrepresentative interpretations of the effect of oral dysbiosis on host responses or even cell death [181]. While the insights from these simplified *in vitro* models remain useful, their oversimplification restricts their use as predictive models for *in vivo* comparisons, with their use in testing therapeutic interventions limited without proper physiological context.

To bridge the gap between oversimplified culture systems and the complex, yet less controllable, *in vivo* environments, there is a clear need to advance *in vitro* models of the oral microbiome. New models should incorporate greater physiological relevance, microbial complexity, and dysbiosis modelling, enabling a deeper understanding of microbiome dynamics in health and disease within a controlled laboratory environment while remaining relevant to real-world applications.

### Computational biology and machine learning

With the massive datasets generated by high-throughput sequencing studies, computational bioinformatics is essential for analysing complex omics data. Building upon this, the development of machine learning (ML) has led to new approaches being utilised for the discovery of new relationships and interactions in microbiome research. Readily available tools, such as scikit-learn, enable new interpretations of existing microbiome data, with classifications and regression facilitating the discovery of biomarkers for both health and disease [182].

For example, Ma et al. (2025) were able to identify the tongue microbiome as a potential marker for the diagnosis of digestive system tumours using models such as generalised linear models, random forest (an algorithm that builds ensembles of decision trees to improve their predictive accuracy) and XGBoost (an efficient gradient boosting framework), which is based on 16S rRNA sequencing from samples of over 1,200 patients [183]. They were able to demonstrate the superior performance of classifiers such as XGBoost, with an AUC of 0.926, compared to others, such as random forest (0.848), suggesting that tongue scrapings may be a non-invasive early biomarker for digestive system tumours. Through conventional statistical bioinformatic analysis, such as LEfSe on taxonomic datasets, the authors were able to decipher *Alloprevotella* and *Prevotella* as enriched in disease while *Neisseria, Haemophilus,* and *Porphyromonas* were dominant in healthy controls. However, while LEfSe identified these specific taxonomic shifts, the ML models were able to leverage complex, non-linear relationships across the entire microbiome community to achieve high diagnostic accuracy. Thus, the study by Ma et al. highlights the utility of ML in accurately predicting disease states where simple taxonomic comparisons alone may fall short. A major limitation of ML involves the ‘black box’ nature of some complex models, where it is difficult to decipher why or how decisions were made by the algorithms, their biological importance, and the specific features (species) that were crucial to the decision-making process.

Similar approaches have already been used in the oral cavity. Pais et al. (2025) utilised machine learning for the classification of healthy and peri-implantitis (PI) samples. The research benchmarked a variety of ML algorithms, including two AI-driven auto ML approaches that generated 100 distinct models. The results demonstrated that these ML models significantly outperformed conventional single microorganism-based classification methods in predicting PI. Notably, models derived from the saliva microbiome, highlighting *Prevotella salivae*, *Streptococcus sanguinis,* and *Fusarium fujikuroi* as key biomarkers, exhibited superior predictive capabilities compared to those from biofilm samples, where *Neurospora crassa*, *Prevotella nigrescens,* and *Alloprevotella tannerae* were prominent. This work underscores the potential of ML in oral microbiome research by demonstrating its ability to develop more accurate, cost-effective diagnostic tools for complex microbiome-related diseases. The study findings, such as the effectiveness of classifiers based on combinations of 2–4 microbes and the viability of saliva sampling, demonstrate highly sensitive disease detection based on microbial signatures showing the usefulness of advanced AI techniques with practical clinical applications in fields such as dentistry and providing a solid foundation for future investigations [184]. By limiting the models to key organisms, the study avoided the ‘black box’ by predetermining the species involved and basing interpretations on the best-performing models.

Beyond identifying taxonomic biomarkers, machine learning can also uncover functional signatures within microbial communities. For instance, Veseli et al. (2025) developed a classifier for IBD based on 33 metabolic modules related to high metabolic independence in the gut microbiome [185]. This classifier effectively distinguished between healthy individuals and those with IBD and monitored recovery post-antibiotic treatment, suggesting that high metabolic independence could be a key indicator of microbial stress adaptation. Such an approach, using ML to identify functional microbial markers, is highly relevant to the oral microbiome, where metabolic processes are crucial in determining health and disease states.

However, a significant challenge remains the interpretability of these complex ML models. To address this, eXplainable AI (XAI) techniques such as SHAP (SHapley Additive exPlanations), which use game theory to explain the output of any machine learning model by attributing importance values to each feature for a particular prediction [186] and LIME (Local Interpretable Model agnostic Explanations), which explains the predictions of any classifier or regressor by approximating it locally with an interpretable model [187] are gaining traction in microbiome applications [188,189]. However, their application in oral microbiome research remains unexplored. Despite their growing use, a considerable gap remains in the quantitative assessment and validation of explanations generated by these XAI techniques, underscoring the need for further research in this area to ensure the reliability of ML-driven insights in microbiome studies [190]. This represents the challenges in utilising ML for research on the oral microbiome, which, coupled with the ‘black box’ nature of some ensemble models, can be challenging to interpret, and the high-dimensional nature of microbiome data can lead to model overfitting highlighting the need for better methodologies in explainable artificial intelligence evaluation [153,190].

## Conclusion

The human oral microbiome is a highly dynamic ecosystem that is foundational to both local and systemic host health. As this review highlights, the shift from a state of eubiosis to dysbiosis not only drives prevalent oral pathologies, such as dental caries and periodontitis, but also facilitates the systemic dissemination of pathogens via the oral–gut axis and bloodstream. Crucially, the oral cavity serves as a substantial yet historically underappreciated reservoir for antimicrobial resistance genes (ARGs). The dense, highly interactive nature of oral biofilms creates an optimal environment for horizontal gene transfer, allowing mobile genetic elements to rapidly disseminate resistance among commensal and pathogenic species alike. This dynamic places the oral resistome firmly at the centre of the global antimicrobial resistance crisis, underscoring the urgent need for robust dental antibiotic stewardship within a broader One Health framework.

To fully comprehend and combat these challenges, our methodological approaches must continue to evolve through integrated, multifaceted research strategies. Conventional culture-based techniques provided the foundational understanding of the oral bacteriome but are inherently limited by the unculturability of fastidious organisms and a lack of community context. The advent of whole-genome sequencing and molecular approaches has revolutionised our capacity to profile microbial diversity and uncover the vast genetic potential of the oral resistome, although these techniques bring forth considerable challenges in data management, standardisation, and comparability.

Furthermore, while these *in silico* discoveries must be grounded in physiological reality through advanced *in vitro* and *in vivo* models, these systems face ongoing challenges regarding ethical considerations, human translatability and the persistent need to balance experimental control with physiological relevance.

Ultimately, the future of oral microbiome research relies on combining the strengths of these diverse methodologies to mitigate their respective weaknesses, spearheaded by advancements in computational biology. Machine learning and artificial intelligence offer unprecedented potential to synthesise highly complex, multi-omics datasets, enabling the identification of novel diagnostic biomarkers and the accurate prediction of antimicrobial susceptibility. By applying these algorithms specifically to genomic data, researchers can begin to map and predict the behaviour of the oral resistome, connecting metabolic pathways and mobile elements directly to their clinical resistance phenotypes. By embracing these advanced analytical tools and addressing challenges surrounding model interpretability and the validation of their outputs (including use of XAI) researchers and clinicians can transition from purely descriptive microbiome studies to predictive and personalised interventions, thereby improving systemic health outcomes and mitigating the spread of antimicrobial resistance.

## Data Availability

Data sharing is not applicable to this article as no new data were created or analysed in this study.

## Generative AI

The authors report generative AI used in their proofreading and final formatting of this manuscript.
